# Maine

**Published:** 1896-08

**Authors:** 


*Maine.* The cattle commissioners of Maine are busy as they can be, with several weeks' work ahead, testing herds of cows reported as tuberculous or on the complaint of local boards of health; much interest and astonishment prevail among the cattle-owners at the tuberculin-test and its accuracy in detecting the diseased ones.

Saco has requested the State Cattle Commissioners to examine the herds supplying that city with milk.

